# Ablation of the Evolutionarily Acquired Functions of the *Atp1b4* Gene Increases Metabolic Capacity and Reduces Obesity

**DOI:** 10.3390/life15071103

**Published:** 2025-07-14

**Authors:** Nikolai N. Modyanov, Lucia Russo, Sumona Ghosh Lester, Tamara R. Castañeda, Himangi G. Marathe, Larisa V. Fedorova, Raymond E. Bourey, Sonia M. Najjar, Ivana L. de la Serna

**Affiliations:** 1 Center for Diabetes and Endocrine Research, College of Medicine and Life Sciences, Health Science Campus, University of Toledo, 3000 Arlington Avenue, Mail Stop1009 Toledo, Toledo, OH 43614, USA; luciarusso.biotech@gmail.com (L.R.); bourey@wustl.edu (R.E.B.); najjar@ohio.edu (S.M.N.); ivana.delaserna@utoledo.edu (I.L.d.l.S.); 2 Department of Physiology and Pharmacology, College of Medicine and Life Sciences, University of Toledo, Toledo, OH 43614, USA; 3 Pulmonology Division, Department of Pediatrics, University of Michigan, Ann Arbor, MI 48109, USA; 4 Department of Cell and Cancer Biology, College of Medicine and Life Sciences, University of Toledo, Toledo, OH 43614, USA; 5 Department of Bioinformatics and Biostatistics, Roswell Park Comprehensive Cancer Center, Buffalo, NY 14263, USA; 6 CRI Genetics LLC, Santa Monica, CA 90404, USA; 7 John T. Milliken Department of Medicine, Division of Endocrinology, Metabolism and Lipid Research, Washington University Medicine, St Louis, MO 63110, USA; 8 Department of Biomedical Sciences, Heritage College of Osteopathic Medicine, Ohio University, Athens, OH 45701, USA; 9 Diabetes Institute, Heritage College of Osteopathic Medicine, Ohio University, Athens, OH 45701, USA

**Keywords:** *ATP1B4*, gene co-option, placental mammal evolution, muscle, metabolism, obesity

## Abstract

In placental mammals, the co-option of vertebrate orthologous *ATP1B4* genes has profoundly altered the properties of the encoded BetaM proteins, which function as bona fide β-subunits of Na,K-ATPases in lower vertebrates. Eutherian BetaM acquired an extended Glu-rich N-terminal domain resulting in the complete loss of its ancestral function and became a skeletal and cardiac muscle-specific component of the inner nuclear membrane. BetaM is expressed at the highest level during perinatal development and is implicated in gene regulation. Here we report the long-term consequences of *Atp1b4* ablation on metabolic parameters in adult mice. Male BetaM-deficient (*Atp1b4−/Y*) mice have remarkably lower body weight and adiposity than their wild-type littermates, despite higher food intake. Indirect calorimetry shows higher energy expenditure (heat production and oxygen consumption) with a greater spontaneous locomotor activity in *Atp1b4−/Y* males. Their lower respiratory exchange ratio suggests a greater reliance on fat metabolism compared to their wild-type counterparts. Consistently, *Atp1b4−/Y* KO mice exhibit enhanced β-oxidation in skeletal muscle, along with improved glucose and insulin tolerance. These robust metabolic changes induced by *Atp1b4* disruption demonstrate that eutherian BetaM plays an important role in regulating adult mouse metabolism. This demonstrates that bypassing the co-option of *Atp1b4* potentially reduces susceptibility to obesity. Thus, *Atp1b4* ablation leading to the loss of evolutionarily acquired BetaM functions serves as a model for a potential alternative pathway in mammalian evolution.

## 1. Introduction

*ATP1B4* genes are present in a single unambiguous copy in all known vertebrate genomes, residing in a highly conserved chromosomal segment. In the human genome, *ATP1B4* is specifically located at Xq24 [1]. The evolutionary history of *ATP1B4* genes is rather unique and represents an instance of orthologous vertebrate gene co-option [2] that radically altered the properties of the encoded BetaM proteins [1]. These proteins in lower vertebrates are authentic Na,K-ATPase β-subunits that form a complex with catalytic α-subunits to function as ion pumps in the plasma membrane. In placental mammals, BetaM proteins acquired distinct properties through structural changes, including the addition of a long N-terminal domain that features an Arg-rich nonapeptide, a characteristic nuclear localization signal [2], as well as two extended Glu-rich clusters [1,3,4,5,6]. These homopolymeric amino acid repeats typically form intrinsically disordered domains that function as flexible molecular recognition elements in numerous signaling proteins and transcription regulators, enabling interactions with a diverse range of binding partners [7,8]. As a result of these evolutionary modifications, eutherian BetaM has entirely lost its ancestral function as a Na,K-ATPase subunit and has instead become a muscle-specific resident of the inner nuclear membrane, with its acquired N-terminal domain exposed to the nucleoplasm [1,5,6,9,10,11,12]. Moreover, in sharp contrast to other structurally related members of the Na,K-ATPase β-subunit family, the polypeptide chain of eutherian BetaM is extremely unstable and highly sensitive to degradation by endogenous proteases [9]. It rapidly disappears in primary culture and is absent in muscle cell lines [1,6,11]. BetaM expression is tightly regulated during development, reaching its highest levels in perinatal skeletal myocytes [5,6,11].

We originally reported that in neonatal skeletal muscle, BetaM directly interacts with a nuclear transcriptional coregulator, SKI-interacting protein (SKIP), and is involved in the regulation of gene expression as exemplified by the induction of the expression of Smad7, which negatively regulates the TGF-β/Smad signaling pathway [1]. We have also shown that expression of exogenous BetaM in cultured muscle cells is sufficient to upregulate the expression of endogenous MyoD, a master regulator of myogenesis, through promoting changes in chromatin structure and enhancing recruitment of SWI/SNF chromatin remodeling enzymes to the DRR region of the MyoD promoter [13].

All these new structural and functional properties acquired through *ATP1B4* gene co-option and a unique pattern of expression strongly suggest that eutherian-specific BetaM functions are essential, and might even be necessary for survival of placental mammals in natural conditions, and that they provide an evolutionary advantage. 

To gain a deeper understanding of the evolutionary advantage conferred by *ATP1B4* gene co-option, we aimed to characterize BetaM function *in vivo*. To this end, we generated the *Atp1b4* knockout mouse model. We found that homozygous *Atp1b4−/−* females have reproductive defects. However, heterozygous *Atp1b4+/−* females and *Atp1b4−/Y* hemizygous males are fertile and generate a progeny that allows the direct comparison of wild-type (WT) and knockout male littermates. The present study offers an *in vivo* characterization of the role of eutherian *ATP1B4* in regulating muscle gene expression, energy balance, and systemic metabolism. The findings demonstrate that *Atp1b4* ablation in mice leads to profound metabolic alterations, which could significantly affect survival in natural environments. These results provide insight into the evolutionary basis for the co-option of eutherian *ATP1B4* genes.

## 2. Materials and Methods

### 2.1. Generation and Maintenance of Atp1b4 Knockout Mice

Gene-targeted ES cells were constructed through the framework of the NIH Knockout Mouse Project (KOMP) using the knockout-first strategy based on inserting a cassette into the intron of the gene that produces a knockout at the RNA processing level [14]. Germline transmitted mice (C57BL/6N) bearing an *Atp1b4*-targeting knockout-first cassette were generated by contract with the NIH KOMP Repository at the University of California, Davis (IKMC Project Report—KOMP-CSD (ID: CSD23729). The *Atp1b4*-targeted ES cells were from C57BL6/J mice. Chimeric mice were backcrossed to the C57BL6/J background for more than 12 generations. Mice were housed under a 12-12-h light–dark cycle (6 a.m. light on) with ad libitum access to tap water and a standard chow (Catalog #2016S, 12 kcal% fat, Harlan Teklad Laboratory Diets, WI, USA). The Institutional Animal Care and Use Committee (IACUC) approved all experimental protocols [14].

### 2.2. Metabolic Parameters

Retro-orbital venous blood was collected at 11:00 a.m. from mice that had been fasted overnight [15]. Blood was collected into heparinized micro-hematocrit capillary tubes (Fisherbrand 22-362-566), immediately chilled on ice, and centrifuged at 3000 rpm, 4 °C, for 30 min, and plasma was collected and stored at −80 °C. Plasma insulin and C-peptide levels were measured using mouse radio-immunoassay kits (Linco Research, St Charles, MO, USA). The intra- and inter-assay coefficients of variation for insulin were 2.7–5.8% and 4.0–11.2% respectively. 

### 2.3. Intraperitoneal Glucose Tolerance Test

Following a 7-h fast (starting at 7 a.m.), conscious mice were injected intraperitoneally (IP) with 50 % dextrose solution (1.5 g/kg body wt), and blood was drawn from their tail vein to measure glucose levels (mg/dL) at 0, 15, 30, 45, 60, and 120 min post-glucose injection. 

### 2.4. Intraperitoneal Insulin Tolerance Test

Mice were fasted for 7 h starting at 7 a.m. Fasted blood glucose was taken from the tail vein by snipping the tail. Awake mice were IP injected with 0.75 U/kg body weight of Human Insulin Novolin (Novo Nordisk, Princeton, NI, USA; NDC 0169-1833-11). Blood glucose was measured from the tail vein at 0, 15, 30, 45, 60, 120, and 180 min post-insulin injection, and glucose levels were expressed as percentages of fasting levels.

### 2.5. Body Composition

Whole-body composition was assessed by Nuclear Magnetic Resonance technology (Bruker Minispec; Billerica, MA, USA).

### 2.6. Energy Balance Measurements

Mice were caged individually in a customized indirect calorimetry system (CLAMS system, Columbus Instrument, Columbus, OH, USA). Energy expenditure (EE), oxygen consumption (VO_2_), CO_2_ production, spontaneous physical activity (SPA), respiratory exchange ratio (RER), and food intake (FI) were monitored simultaneously over 3 consecutive days following a 2-day acclimation period. Measurements were taken every 20 min at a flow rate of 0.5 L/min. 

### 2.7. Ex Vivo Palmitate Oxidation

As previously carried out [16], mice (4 months of age, n = 5) were fasted overnight and anesthetized before their soleus and gastrocnemius muscles were removed, weighed, mixed, homogenized, and injected into a sealed beaker at 30 °C to initiate the reaction in incubation buffer containing 0.2 mmol/L [1-14C]palmitate at 18,500 Bq/mL in the presence of 2 mmol/L L-carnitine, 0.1 mmol/L malic acid, 2 mmol/L ATP, and 0.05 mmol/L coenzyme A. After 45 min, the reaction was terminated with glacial acetic acid. Radioactive CO_2_ was trapped in a well, which was suspended above the medium and filled with benzothonium hydroxide, and counted by liquid scintillation (CytoCint; MP Biomedicals, Solon, OH, USA).

### 2.8. Protein Isolation and Western Blotting

Total protein was isolated in RIPA buffer (50 mM Tris-HCl, pH 7.5, 0.1% Triton-X-100, 0.5% Nonidet P-40, 0.15 M NaCl, 1 mM EDTA, 1 mM Na_3_VO_4_, 0.5 mM PMSF, 100 μL protease inhibitor (Sigma, St. Louis, MO, USA)) [1]. Protein concentration was measured by a BCA protein assay (Pierce Biotechnology, Rockford, IL, USA). Equal amounts of protein were loaded and subjected to electrophoresis on 10% SDS-PAGE gels. Proteins were transferred to PVDF membranes. Membranes were blocked with 5% nonfat milk, washed, incubated with primary anti-BetaM antibody [9], washed, and then incubated with HRP-linked secondary antibody. Blots were then stripped and silver-stained [17]. 

### 2.9. Semi-Quantitative and Quantitative Real Time-PCR (qRT-PCR)

Mice were sacrificed, and hindleg muscle tissue was collected, snap-frozen in liquid nitrogen, and stored at −80 °C for measurement of mRNA content. Total RNA was extracted from frozen tissue samples using PerfectPure RNA Tissue Kit–50 (5 PRIME - 2900304; 2900317- Gentra Systems, Minneapolis, MN, USA), and its quantity and purity were determined by absorbance at 260 and 280 nm. cDNA templates for RT-PCR were synthesized using 1 μg of total RNA, 10X DNase Reaction Buffer, DNase 1 Amp Grade, dNTPs, Random Primers, and Superscript III (Invitrogen, Waltham, MA, USA). Semi-quantitative PCR using BetaM and GAPDH primers was performed as previously reported [13]. qRT-PCR was performed using SYBR Green Master Mix (Smart Bioscience, Maumee, OH, USA) and amplified on an ABI StepOnePlus Real-Time PCR System (Applied Biosystems, Foster City, CA, USA). The expression level of each experimental gene was normalized to the concentration of the housekeeping gene 18S in each sample. Primers for qRT-PCR are listed in Table 1. 

### 2.10. Statistical Analysis

Quantitative data are presented as mean ± standard error of the mean (SEM). Values were analyzed for statistically significant differences using one-way ANOVA and a post hoc Tukey test or two-tailed unpaired *t*-test. *p* < 0.05 was considered statistically significant (Graph Pad Prism software 5.0).

## 3. Results

### 3.1. Construction of the BetaM Knockout Mouse

**Figure 1:**
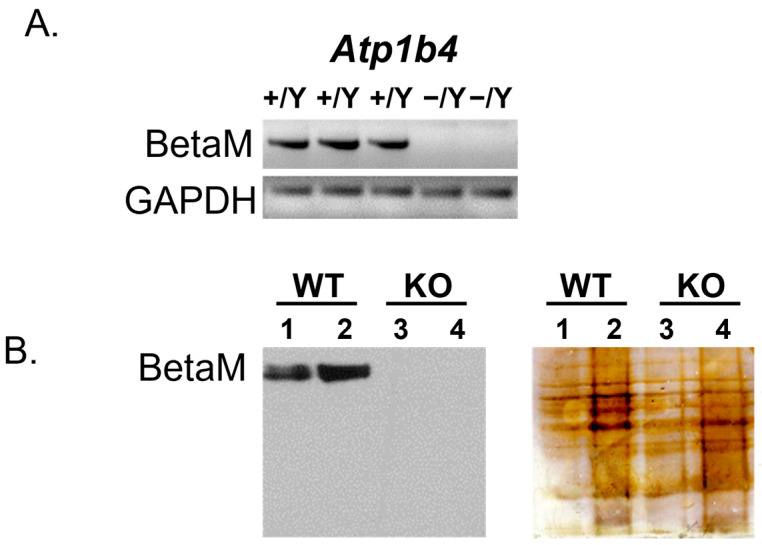
Ablation of BetaM in hemizygous males. (**A**) Semi-quantitative RT-PCR analysis (35 cycles) of RNA from tongue skeletal muscle of 3-day-old wild-type (WT) and knockout (KO) littermates using primers complementary to exons 3 and 5 of Atp1b4. GAPDH is shown as a control. The PCR product was run on an agarose gel. (**B**) (Left) Immunoblot of protein from pooled tongue and hindlimb muscles of 3-day-old WT and KO littermates using BetaM antibodies. (Right) Silver staining of the blot shows equal protein loading. Total protein loaded: 10mg (lanes 1 and 3) and 30mg (lanes 2 and 4).

Chimeric mice with *Atp1b4* ablation were propagated on a C57BL6/J genetic background. As shown in Figure 1A, BetaM expression was lost at the mRNA level, and there was a corresponding loss of the BetaM protein (Figure 1B) in hemizygous males carrying the knockout-first cassette. Initial characterization of *Atp1b4* knockout (KO) mice revealed that homozygous *Atp1b4−/−* females have reproductive defects. The majority of females are sterile, and the rest have fewer pregnancies with only three or four fetuses. Heterozygous *Atp1b4−/+* females and *Atp1b4−/Y* hemizygous males are fertile and generate progeny that allow a direct comparison of WT and KO male littermates. The survival rate of *Atp1b4−/Y* males and *Atp1b4−/−* females is 2–3 times lower (*p* < 0.0003, Chi-square test) than that of male wild-type (WT) and *Atp1b4−/+* female littermates, respectively.

### 3.2. Mice Deficient in Atp1b4 Exhibit Reduced Body Weight and Adiposity

**Figure 2:**
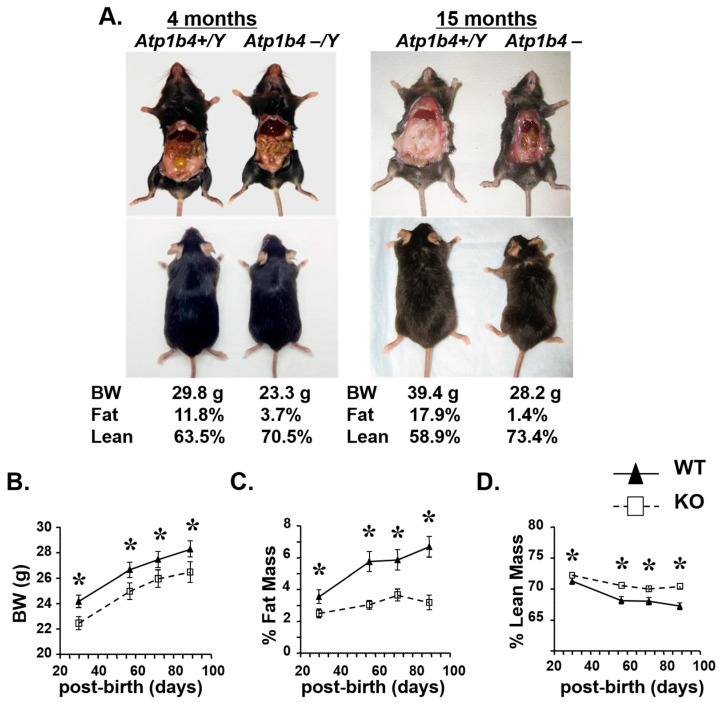
Atp1b4 KO mice have reduced body weight and altered body composition. (**A**) Representative pictures of 4-month-old (**left**) and 15-month-old (**right**) *Atp1b4+/Y* wild-type and *Atp1b4−/Y* KO littermates fed with a regular chow diet beginning at weaning. (**B**) Body weight, (**C**) % fat mass, and (**D**) % lean mass were assessed. (n = 10/genotype) after weaning at the indicated time points after birth. Values are expressed as mean ± SEM. * *p* ≤ 0.05 versus *Atp1b4+/Y*.

Birth weight has been shown to be associated with future risk of human adult metabolic dysfunction, including type 2 diabetes, obesity, and cardiovascular disease [18,19]. Therefore, we analyzed the effect of *Atp1b4* deletion on metabolism in adult male mice. KO mice were smaller and had significantly less visceral fat at 4 months of age compared to age-matched WT mice (Figure 2A, left). The difference in fat accumulation between KO and WT mice became even more pronounced by 15 months of age (Figure 2A, right). KO mice exhibited a 20% reduction in body weight (Figure 2B) and a 70% reduction in fat mass (Figure 2C) with a reciprocal increase in lean mass (Figure 2D) compared with control *Atp1b4+/Y* (WT) mice.

**Figure 3:**
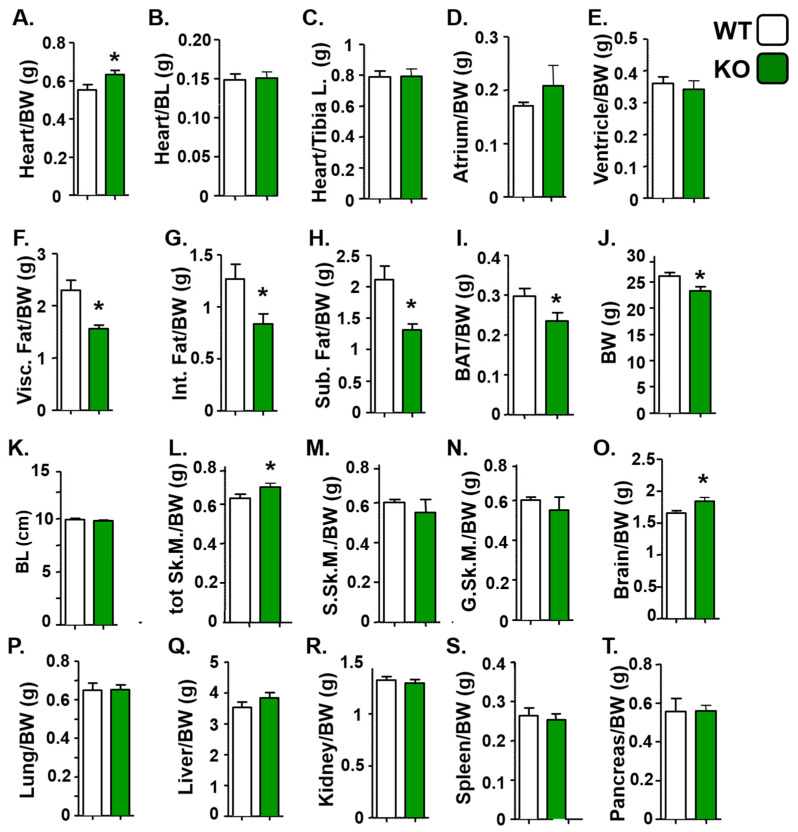
Atp1b4 KO mice exhibit a reduction in adiposity. Four-month-old *Atp1b4+/Y* (WT) and *Atp1b4−/Y* (KO) mice (n = 10/genotype) fed a regular chow diet were sacrificed, and the indicated tissues were dissected and weighed. Tissue mass is shown relative to body weight (**A**,**D**–**I**,**L**–**T**), body length (**B**) or tibia length (**C**) and represented as mean ± SEM. * *p* ≤ 0.05 versus *Atp1b4+/Y*. BW = body weight (**J**), BL = body length (**K**), Int. Fat = internal fat, Visc. Fat = visceral fat, Sub. Fat = subcutaneous fat, BAT = brown adipose tissue, G. Sk.M = gastrocnemius skeletal muscle, S. Sk.M = soleus skeletal muscle.

We conducted additional analyses to characterize distinct physical attributes in KO mice. KO mice exhibited a slight increase in skeletal and cardiac mass relative to body weight (Figure 3A), but showed no significant changes in heart mass relative to body length (Figure 3B) or tibia length (Figure 3C), nor were there specific changes in atrial-to-body-weight (Figure 3D) or ventricular-to-body-weight ratios (Figure 3E). KO mice exhibited markedly altered body composition, primarily due to a reduction in all major adipose depots—including visceral adipose tissue (Figure 3F), internal adipose tissue (Figure 3G), subcutaneous adipose tissue (Figure 3H), and brown adipose tissue (Figure 3I)—accompanied by a significant decrease in overall body weight (Figure 3J), but no significant change in body length (Figure 3K). Although we observed a slight increase in total skeletal muscle mass relative to body weight in KO mice (Figure 3L), this increase was likely distributed evenly throughout different muscle groups, as there were no significant changes in the mass of the soleus (Figure 3M) or gastrocnemius muscles (Figure 3N). Similarly, while brain mass relative to body weight showed a modest increase (Figure 3O), the masses of other organs remained unchanged (Figure 3P–T). These findings suggest that the loss of BetaM leads to reduced body weight primarily through a marked decrease in fat accumulation, without reducing the mass of other tissues.

### 3.3. Elevated Energy Expenditure in Atp1b4-Deficient Male Mice 

**Figure 4:**
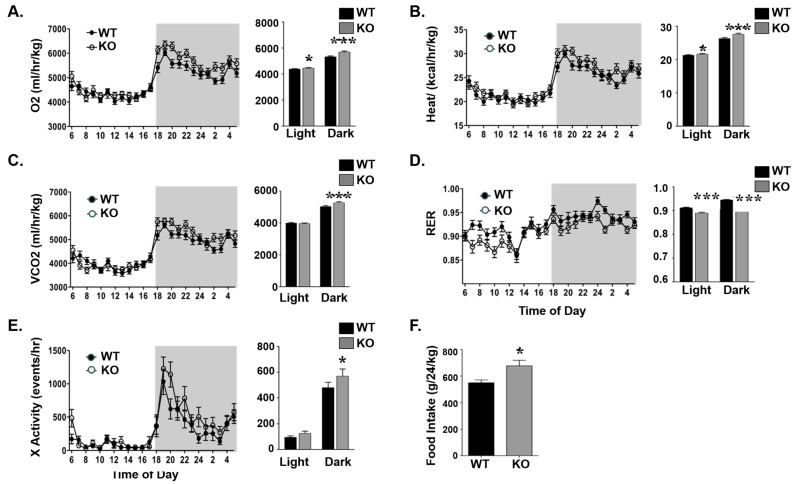
Increased energy metabolism in Atp1b4 KO mice. Three-month-old male *Atp1b4+/Y* (WT) and *Atp1b4−/Y* (KO) mice were individually caged (n = 8/genotype), given free access to food ad libitum, and subjected to indirect calorimetry analysis in a 24 h period for 5 days to assess (**A**) O_2_ consumption (mL/h/kg lean mass), (**B**) heat generation or energy expenditure (kcal/h/kg lean mass), (**C**) VCO_2_ production (mL/h/kg lean mass), (**D**) respiratory exchange ratio (RER), (**E**) spontaneous activity, and (**F**) food intake. Values are expressed as mean ± SEM. * *p* ≤ 0.05, *** *p* < 0.005 versus *Atp1b4+/Y*.

We next examined the mechanisms underlying the lower adiposity in KO mice. Indirect calorimetry analysis showed that 3-month-old KO mice maintained higher energy expenditure [O_2_ consumption (Figure 4A), heat generation (Figure 4B)] and CO_2_ production (Figure 4C). Moreover, they had a lower respiratory exchange ratio (RER), suggesting the preferred use of fat rather than carbohydrates as an energy source (Figure 4D). Additionally, KO mice showed increased spontaneous locomotor activity (Figure 4E) and increased food consumption (Figure 4F) compared with their control counterparts.

### 3.4. Elevated Fatty Acid β-Oxidation and Reduced Lipid Accumulation in Atp1b4 KO Mice

**Figure 5:**
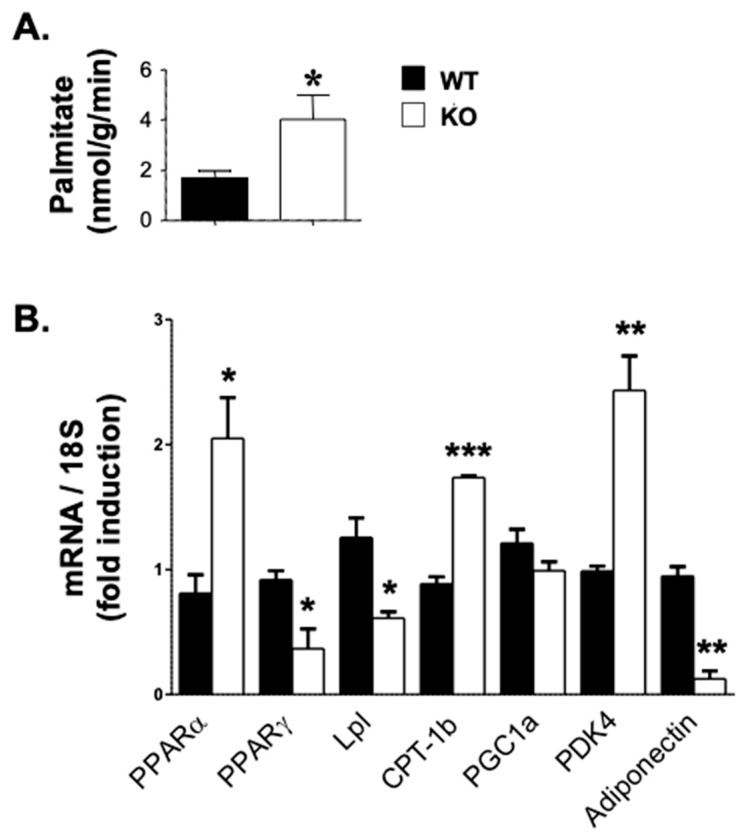
Elevated fatty acid β-oxidation in skeletal muscle of Atp1b4 KO mice. (**A**) Fatty acid β-oxidation in skeletal muscle in 4-month-old male *Atp1b4+/Y* (WT) and *Atp1b4−/Y* (KO) mice fed a regular diet (n = 6/genotype). (**B**) qRT-PCR analysis of genes involved in lipid homeostasis normalized to 18S RNA. Values are expressed as mean ± SEM. * *p* ≤ 0.05, ** *p* < 0.01, *** *p* < 0.005 versus *Atp1b4+/Y*.

Consistent with increased energy expenditure, fatty acid β-oxidation was significantly increased in the skeletal muscle of KO mice compared to their WT controls, as assessed by [^14^C] palmitate oxidation [16] (Figure 5A) and elevated mRNA levels of peroxisome proliferator-activated receptor alpha (PPARα), a major regulator of lipid metabolism. The activation of PPARα promotes the uptake, utilization, and catabolism of fatty acids by transcriptional upregulation of the expression of genes involved in fatty acid transport, fatty acid binding and activation, and peroxisomal and mitochondrial fatty acid β-oxidation [20]. Some of the genes regulated by PPARα include Pyruvate Dehydrogenase Kinase 4 (PDK4), which phosphorylates and inhibits the pyruvate dehydrogenase complex, thus contributing to the regulation of glucose metabolism [21]. PPARα also upregulates the expression of Carnitine Palmitoyltransferase 1B (CPT-1b), which is required for the net transport of long-chain fatty acyl-CoAs from the cytoplasm into the mitochondria [22]. Interestingly, PDK4 and CPT-1b were upregulated by 2.5- and 2-fold, respectively, in the muscle of KO mice. In contrast, the mRNA levels of genes implicated in lipid accumulation were reciprocally repressed in KO mice (Figure 5B). These include PPARγ, which upregulates fatty acid storage and glucose metabolism [23]. When activated, PPARγ stimulates lipid uptake and adipogenesis by fat cells and the upregulation of adipogenic genes, such as lipoprotein lipase (Lpl). As a homodimer with a triglyceride hydrolase activity, Lpl serves as a ligand/bridge factor in receptor-mediated lipoprotein uptake [24]. Another PPARγ-targeted gene is adiponectin, which circulates in the plasma to regulate metabolic processes including glucose homeostasis and fatty acid β-oxidation [25]. The mRNA levels of both Lpl and adiponectin were lower in the skeletal muscle of KO than WT mice (Figure 5B).

**Figure 6:**
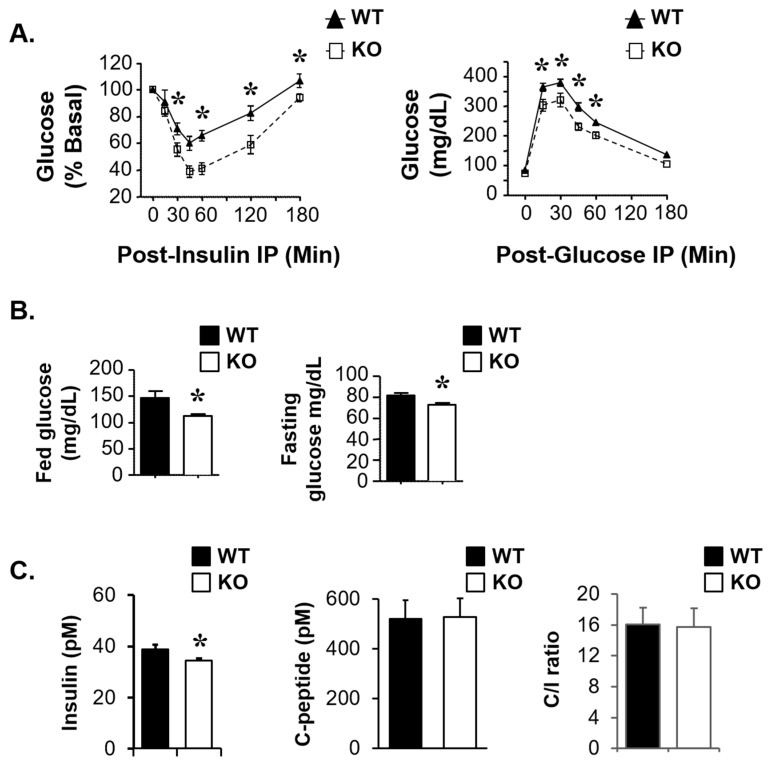
**Effect of Atp1b4 deficiency on biochemical parameters in 4-month-old male mice.** (**A**) The tail vein glucose level was determined in response to IP injection of insulin (**left**) and glucose (**right**) to measure insulin and glucose tolerance, respectively. (**B**) Fed (**left**) and fasting (**right**) blood glucose levels. (**C**) Plasma insulin (**left**), C-peptide (**middle**), and C-peptide-to-insulin (C/I) molar ratio (**right**) were determined in 4-month-old male *Atp1b4+/Y* and *Atp1b4−/Y* male mice fasted overnight (n = 10/genotype). Values are expressed as mean ± SEM. * *p* ≤ 0.05versus *Atp1b4+/Y*.

We then examined whether Atp1b4 gene deletion affects insulin sensitivity and glucose homeostasis. As Figure 6A (left graph) shows, 4-month-old KO mice exhibited better tolerance to exogenous insulin than their WT counterparts. Together with lower random blood glucose levels (Figure 6B, left) and serum insulin levels (Figure 6C, left), this indicates increased insulin sensitivity in KO relative to WT mice. KO mice also showed better tolerance to exogenous glucose (Figure 6A, right) and lower fasting blood glucose levels (Figure 6B, right). Consistent with the metabolic effects being primarily driven by improved insulin sensitivity, insulin secretion, as indicated by surrogate serum C-peptide levels (Figure 6C, middle), and hepatic insulin clearance, assessed by the steady-state C-peptide/insulin ratio (Figure 6C, right), did not change in KO relative to WT mice. Taken together, these data demonstrate that the deletion of BetaM improves glucose and insulin metabolism and action.

## 4. Discussion

The *ATP1B4* genes, members of the X,K-ATPase β-subunit gene family, are a rare example of orthologous vertebrate gene co-option [26]. This evolutionary phenomenon has profoundly altered the functional properties of the encoded BetaM proteins, significantly transforming their structure and expression patterns [1,4,5,6,9,10,11]. The expression of eutherian BetaM is tissue-specific, with the highest level being in skeletal muscle, followed by the heart and then the skin [4,5,6,9]. In skeletal muscle, BetaM expression is temporally regulated; it is robustly induced in the last quarter of pregnancy, remains high in neonatal muscle, and is undetectable in adult mouse muscle. This pattern of expression suggests that the function of BetaM is linked to myogenic developmental processes [4,5,6,9,10,27,28,29]. We previously showed that BetaM interacts with the nuclear transcriptional coregulator Ski-interacting protein (SKIP) in neonatal muscle, influencing the activity of TGF-β-responsive reporters. BetaM binds to the promoter and induces the transcription of Smad7, a critical negative regulator of TGF-β/Smad2-3 signaling [1]. These findings provided the first experimental evidence that eutherian BetaM functions as a transcriptional regulator. We recently reported that BetaM binds to the MyoD promoter *in vivo* and found that exogenously expressed BetaM enhanced MyoD expression in cultured myoblasts, indicating a regulatory role in myogenesis [13]. BetaM promoted histone modifications and recruited the SWI/SNF chromatin remodeling subunit, BRG1. These results indicate that eutherian BetaM regulates muscle gene expression by promoting changes in chromatin structure.

To further investigate the *in vivo* function of BetaM, we generated a whole-body *Atp1b4* knockout mouse model. Surprisingly, the absence of BetaM in neonatal hemizygous male mice significantly affects whole-body metabolism, as shown in the current studies. Because BetaM expression is restricted to neonatal muscle, this demonstrates that the loss of BetaM in muscle impacts whole-body metabolism. Skeletal muscle, a major site for the regulation of fatty acid and glucose metabolism, plays a significant role in the control of obesity, diabetes, and cardiovascular disease, and is itself regulated by whole-body metabolism [30,31,32]. As the largest organ that also responds to insulin, skeletal muscle is responsible for approximately 80% of glucose uptake from the circulation. Increased energy supply and a sedentary lifestyle cause lipid accumulation and metabolic alterations in skeletal muscle that impede glucose regulation and contribute to the development of insulin resistance [33,34]. Since skeletal muscle releases myokines that influence whole-body metabolism and insulin sensitivity by modulating inflammation, it is possible that the loss of BetaM alters the production or secretion of these factors. Moreover, BetaM loss had no impact on insulin secretion or clearance, consistent with the lack of BetaM expression in pancreatic β-cells and hepatocytes, respectively [35,36]. Thus, insulin and glucose homeostasis were maintained at a normal state in *Atp1b4−/Y* KO male mice, in concordance with insulin sensitivity in these mice. 

Although BetaM expression gradually declines after birth and becomes nearly undetectable in adult mouse skeletal muscle [5,6,11], the activation of *ATP1B4* was detected in the superficial fascia tissue of patients with incisional hernias, along with the activation of inflammatory pathways [37]. This suggests that specific physiological conditions, such as injury, can trigger the re-initiation of its expression. Another study linked high *Atp1b4* mRNA levels to the remarkable adaptation of Tibetan pigs to extreme environmental conditions, including low oxygen levels, severe cold, and intense UV radiation [38]. Collectively, these findings suggest that BetaM expression is induced in adults under stress and may influence muscle metabolism, potentially impacting whole-body metabolic regulation. Currently, we are investigating the mechanisms that regulate BetaM expression and its impact on overall metabolism in adults. Nonetheless, our findings provide *in vivo* evidence that BetaM plays a critical regulatory role in metabolism, underscored by the unexpected discovery that BetaM promotes fat accumulation. This function of BetaM may contribute to the rising prevalence of obesity.

A limitation of our study is the exclusive focus on male mice, which prevents us from determining whether BetaM loss similarly affects metabolism in females. This choice was driven by the reduced fertility of homozygous female knockout mice and the heterogeneous expression of BetaM in heterozygous females, due to the gene’s location on the X chromosome and its susceptibility to random X-inactivation. Historically, many mouse models of obesity have excluded females, resulting in a lack of reliable data on sex-specific metabolic responses and their translational relevance to humans. Notably, studies that have included both sexes have revealed striking differences [39,40]. For example, one study found that male and female C57BL/6J mice respond differently to dietary manipulation in terms of weight gain, food intake, motor activity, energy expenditure, and β-cell adaptation, with females displaying milder phenotypic changes [39]. These findings underscore the importance of including both sexes in metabolic research. Future studies will therefore examine the effects of BetaM loss in both males and females, under various dietary conditions [39,40]. 

There has been increasing recognition of the evolutionary origins of obesity. Several hypotheses have been put forth to explain how past selective pressures have shaped metabolic traits that contribute to excess fat accumulation in modern environments. For example, the “Thrifty Gene” Hypothesis proposes that genes promoting efficient fat storage were advantageous in ancestral times by allowing individuals to survive famine, but in modern societies, with abundant food and sedentary lifestyles, lead to obesity [41]. The evolution of placental mammals (eutherians) led to the birth of fully developed young that are metabolically independent of the mother. Consequently, eutherians have adopted distinct metabolic parameters such as higher metabolic rates and alterations in thermogenesis compared with non-eutherian species [42,43]. These evolutionary changes potentially shape pathways regulating energy storage and utilization that may contribute to modern obesity trends. Our findings suggest that the acquired function of BetaM in eutherians, analogous to “thrifty” genes, establishes it as an important regulator of metabolic processes. This suggests a dual role for BetaM: enhancing survival while simultaneously increasing susceptibility to obesity.

A small portion of the initial studies were reported in a meeting abstract [44].

## Figures and Tables

**Table 1:** Primer sequences used for quantitative RT-PCR.

Primer	Forward Sequence	Reverse Sequence
18S	TTCGAACGTCTGCCCTATCAA	ATGGTAGGCACGGCGACT
Ppar-a	TGCTGGTATCGGCTCAATAA	TCCTGCCACTTGCTCACTAC
Ppar-g	AGATCATCTACACGATGCTGGCCT	ATAAAGTCACCAAAGGGCTTCCGC
Lpl	AAGGTCAGAGCCAAGAGAAGCA	CCAGAAAAGTGAATCTTGACTTGGT
Cpt-1b	CAGCGCTTTGGGAACCACAT	CACTGCCTCAAGAGCTGTTCTC
Pgck-1a	AACAAGCACTTCGGTCATCCCTG	TTACTGAAGTCGCCATCCCTTAG
Pdk4	TTTCTCGTCTCTACGCCAAG	GATACACCAGTCATCAGCTTCG
Adiponectin	GGCCGTTCTCTTCACCTACG	TGGAGGAGCACAGAGCCAG

## Data Availability

No new data were created.
